# Diagnostic significance of posterior pharyngeal lymphoid follicles in seasonal influenza

**DOI:** 10.1002/jgf2.475

**Published:** 2021-06-24

**Authors:** Koki Kato

**Affiliations:** ^1^ Madoka Family Clinic Fukuoka Japan; ^2^ Hokkaido Centre for Family Medicine Academic and Research Centre Sapporo Japan

## CONFLICT OF INTEREST

The author has stated explicitly that there are no conflicts of interest in connection with this article.

To the Editor,

In their recently published study, Takeoka et al.^1^ investigated helpful findings for diagnosing seasonal influenza. They conducted a single‐center case‐control study including 176 cases and 38 controls and demonstrated that the odds ratio of posterior pharyngeal lymphoid follicles in patients with seasonal influenza was 2.71 (95% confidence interval [CI], 1.17–6.28) and that the specificity was 0.711 (95% CI, 0.541–0.846). They then concluded that the finding "would be useful when patients with influenza‐like symptoms were false‐negative for the rapid antigen test." However, I have some concerns about the interpretation and presentation of the results of this study.

First, this study causes a high risk of observer bias. This is because the observers could obtain the rapid antigen test results almost at the time of physical examination. The observers would be affected to record a positive finding of posterior pharyngeal lymphoid follicles when the test results were positive.

Second, the control group did not include the younger population, who would have a higher prevalence of posterior pharyngeal lymphoid follicles not due to influenza. The age of the control group was higher than that of the case group (27 vs 22, *p* = 0.08). Posterior pharyngeal lymphoid follicles can be observed in patients infected with, for example, mycoplasma,^2^ adenovirus,^3^ and echovirus.^3^ Those infections are prevalent in the younger population. Thus, they should have adjusted the multivariate logistic analysis by the age variable.

Lastly, the conclusion was beyond the scope of the research findings. The specificity of posterior pharyngeal lymphoid follicles may not be sufficiently high to confirm the diagnosis of influenza in conditions with negative rapid antigen test which reduce the post‐test probability.

This study is informative for clinicians, suggesting a valuable finding for diagnosing seasonal influenza. However, considering the above, I have concern that there was an overestimation of the odds ratio and diagnostic significance of the posterior pharyngeal lymphoid follicles for diagnosing seasonal influenza. I think it would be better to inform readers of the above limitations and concerns.
